# Transcriptome profiling of venom gland from wasp species: de novo assembly, functional annotation, and discovery of molecular markers

**DOI:** 10.1186/s12864-020-06851-0

**Published:** 2020-06-24

**Authors:** Junjie Tan, Wenbo Wang, Fan Wu, Yunming Li, Quanshui Fan

**Affiliations:** 1 General Hospital of Western Theater Command, Chengdu, 610021 China; 2 CDC of Western Theater Command, PLA, Chengdu, 610021 China

**Keywords:** Venom gland, Wasps, Transcriptome, Simple sequence repeats, Single nucleotide polymorphisms

## Abstract

**Background:**

*Vespa velutina*, one of the most aggressive and fearful wasps in China, can cause grievous allergies and toxic reactions, leading to organ failure and even death. However, there is little evidence on molecular data regarding wasps. Therefore, we aimed to provide an insight into the transcripts expressed in the venom gland of wasps.

**Results:**

In our study, high-throughput RNA sequencing was performed using the venom glands of four wasp species. First, the mitochondrial cytochrome C oxidase submit I (COI) barcoding and the neighbor joining (NJ) tree were used to validate the unique identity and lineage of each individual species. After sequencing, a total of 127,630 contigs were generated and 98,716 coding domain sequences (CDS) were predicted from the four species. The Gene ontology (GO) enrichment analysis of unigenes revealed their functional role in important biological processes (BP), molecular functions (MF) and cellular components (CC). In addition, c-type, p1 type, p2 type and p3 type were the most commonly found simple sequence repeat (SSR) types in the four species of wasp transcriptome. There were differences in the distribution of SSRs and single nucleotide polymorphisms (SNPs) among the four wasp species.

**Conclusions:**

The transcriptome data generated in this study will improve our understanding on bioactive proteins and venom-related genes in wasp venom gland and provide a basis for pests control and other applications. To our knowledge, this is the first study on the identification of large-scale genomic data and the discovery of microsatellite markers from *V. tropica ducalis* and *V. analis fabricius*.

## Background

*Vespa velutina* is native to Indochinese regions, Indonesia, and Taiwan [1, 2]. It was spread to France and Europe in 2004 [3] and was first recorded in South Korea in 2003 [4]. *Vespa mandarinia smith* is found in Korea, Japan, China, and Europe [5]. *Vespa tropica ducalis* has been recorded in India, Japan, France, Nepal, and China [6]. *Vespa analis* Fabricius is mainly distributed in northern India, China, South Korea, Siberia, and Sumatra [7]. These wasps belong to the eusocial groups and live in dense bushlands or mountainous regions where they nest and prey on honeybees, insects, and even other wasps [8, 9]. The wasps are the main carnivorous insects, which can effectively hunt and eliminate agricultural and forest pests such as *Heliothis armigera*, *Artogeia rapae* and locusts [10]. Their hunting habits can serve as an alternative efficient way for biological protection of some crops [11]. Therefore, the use of wasps to control pests can reduce pesticides-induced environmental pollution, with good economic and ecological benefits. However, due to their aggressiveness and activeness, wasps can also cause serious damage to farm industries, especially apiaries, and human health, particularly in allergic people, and can occasionally even be deadly [12]. Recent studies have shown that approximately 1300 people in New Zealand may seek medical services for wasp stings each year [13]. *V. velutina*, one of the most aggressive and fearful wasps in China, can cause grievous allergies and toxic reactions, leading to organ failure and even death [14]. The wasp sting can only be symptomatic and there is no specific treatment. Developing antivenin-like anti-bee venom has a good application prospect.

The commercial value of vespa amino acid mixtures (VAAM) is the economic significance of these species. VAAM has been shown to increase endurance during exercises such as swimming [15], decrease lactate accumulation and increase glucose concentration after running in mice [16]. VAAM ingestion has been shown to increase aerobic fitness and decrease intra-abdominal fat in women who exercise regularly [17]. However, wasp sting can cause skin hemorrhage and potentially lead to allergic reactions resulting in organ failure [18, 19]. Many bioactive peptides and macromolecular proteins, including enzymes, allergens, and toxins, are abundant in the venom of these wasps [20–22].

Currently, there are few studies on molecular data regarding wasps. Therefore, it is necessary to conduct more studies on gene sequences and regulation mechanisms to contribute to the in-depth understanding of their venom components and developing therapeutics for wasp stings. At present, the transcriptome of *V. velutina* has been deciphered, and related genes in the venom gland, such as the putative toxin sequences, have been revealed [14]. The mitochondrial genome sequence of *V. mandarinia smith* has been reported, and the phylogenetic analysis of this wasp was performed based on this information [23]. Moreover, the transcriptome profile of *V. mandarinia smith* was obtained using Illumina sequencing [5]. However, no genome or transcriptome information is as yet available for *V. tropica ducalis* and *V. analis fabricius*. Protein and peptide compounds are regarded as the bioactive substances in the wasp venom, and 398 wasp venom-related proteins were annotated in the UniProt database including mastoparan-like peptide, tachykinin-like peptide, vespin, melittin, venom protein and peptide, phospholipase, polybine, dominulin, and sodium channel subunit (https://www.uniprot.org/). These venom proteins can cause cell degranulation owing to the hemolytic activity [24] or via other relative physiological processes [22, 25, 26]. Despite this information, the genetic and molecular data are still limited and insufficient for high-throughput functional analysis to reveal the mechanisms associated with predation, breeding, communication, and other behaviors of these wasps. Furthermore, for exploring the toxicology of wasp injuries and pharmacology of wasp sting therapy, more information on the whole genome or transcriptome of these species is required to unravel rare gene regulators, new gene mutants, alternative splicing mechanisms, and microsatellite markers, which can promote further research on the target functional genes.

Wasp insects have many similarities in phenotype and morphology, which renders species-specific identification difficult. However, verification of the specific venom is significant for the clinical treatment of wasp injury. DNA barcoding is reported to be an efficient tool for species identification in both animals and plants [27–29]. Snake venom was successfully separated using the mitochondrial 12S gene [30] and the COI barcode [31]. This method was applied for the verification of spider and ant species [32–34]. Furthermore, DNA barcoding has also been reported for the identification and taxonomic classification of the wasp subfamily [35–37].

Whole DNA and RNA sequencing strategies have been successfully applied to address the genomic challenges in eusocial insects. In particular, transcriptome-wide studies have provided insights into caste systems, and the phenotypic plasticity of the genome has been studied in the facultatively eusocial bee, *Megalopta genalis* [38], *Apis cerana cerana* [39] and bumblebee, *Bombus terrestris* [40] by using conventional and high-throughput sequencing technologies. Next-generation sequencing (NGS) technology and the rapid development of high-throughput platforms have allowed the sequencing of non-model organisms.

In this study, we isolated RNA from the venom glands of four different species of Asian giant hornets, *V. velutina*, *V. mandarinia smith*, *V. tropica ducalis*, and *V. analis fabricius* and constructed a cDNA library for whole-transcriptome sequencing by using the latest Illumina platform HiSeq 4000. The sequencing raw reads were preprocessed to obtain quality reads and subsequently processed to obtain assembled contigs and unigene clusters using the Trinity de novo assembler. To our knowledge, this is the first study on the identification of large-scale genomic data and the discovery of microsatellite markers from *V. tropica ducalis* and *V. analis fabricius*.

## Results

### DNA barcoding and tree-based identification

After amplifying the COI gene-specific sequence of eight individuals from the four species, NJ-tree analysis based on the Kimura 2 Parameter distance (K2P) revealed the distinctive difference in COI sequences between the seven groups and estimated the intergeneric and intraspecific sequence divergences.

**Fig. 1:**
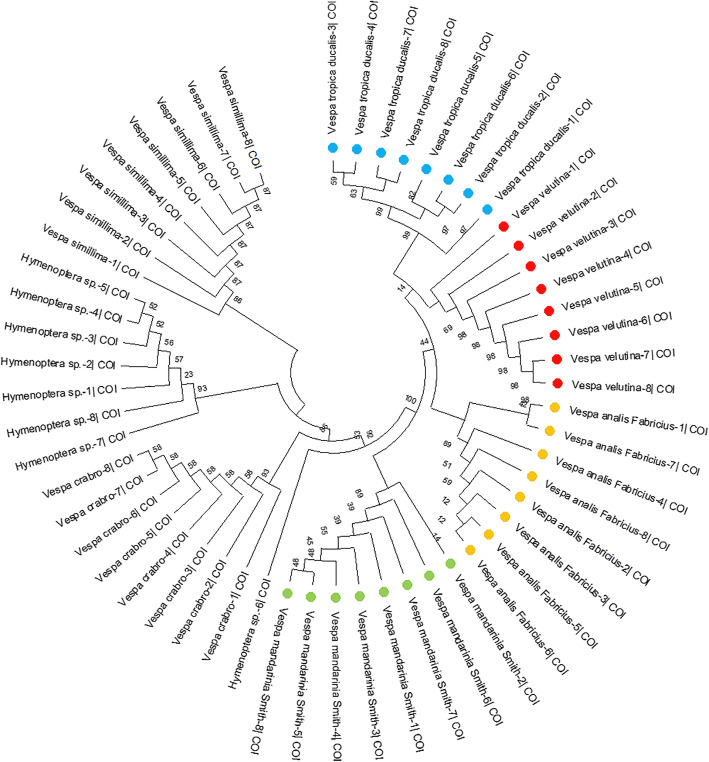
Neighbor-joining tree of wasp samples based on COI gene. Orange color refers to *V. velutina*; green refers to *V. analis fabricius*; blue refers to *V. tropica ducalis*; red refers to *V. mandarinia smith*. Outgroup species: *Vespa simillima simillima, Vespa crabro flavofasciata* and *Hymenoptera sp*.

Based on COI sequence identification, the NJ tree revealed the unique lineage of these individuals, and the clustering information clarified the differences and similarities in the molecular sequences (Fig. 1 and Additional file 1). Seven different wasps were clearly distinguished. Notably, *V. analis fabricius* 1 and *V. analis fabricius* 7 were the factors that contribute to the group sequence variation of the other six unanimous individuals, indicating the occurrence of probable mutation or evolution process in this species (*V. analis fabricius*). Therefore, DNA barcoding could possibly be applied for the identification of wasps with similar or unknown characteristics based on the COI sequence identification. The results also indicated that these species were distinct and could be used for subsequent comparison studies.

### RNA-Seq and de novo assembly of wasp transcriptome

The cDNA libraries from the venom glands of 12 wasp individuals were sequenced using the Illumina platform. 452,427,244 clean and high-quality reads were obtained by deleting redundant transcripts, and the filtering rates of the sequencing reads ranged from 87.75 to 91.70% (Additional file 2). The clean and high-quality reads of RNA-Seq from the four wasp species were assembled into 127,629 contigs corresponding to 323,495,099 base pairs (bp) in total (Table 1). The maximum contig length was 28,994 bp, and the minimum was 301 bp, with an average length of 2534 bp and an N50 value of 3163 bp (Table 1). In addition, the number of contigs differed across the four species, ranging from 65,229 to 76,458, where the highest number was detected in *V. mandarinia smith*, possibly indicating more genome information (Table 1).

**Table 1:** The statistics of the sequencing data after quality trimming

Item	Samples				Total
	VV	VAF	VTD	VMS	
Number of contigs	73,672	65,229	70,510	76,458	127,629
Number of characters (bp)	90,906,977	84,883,428	84,763,580	97,906,925	323,495,099
Average Length (bp)	1234	1302	1202	1280	2534
Minimum Contigs Length	301	301	301	301	800
Maximum Contigs Length	27,683	27,018	24,129	28,994	36,048
N50 Length	2026	2154	1957	2086	3163
Median Length	694	728	677	726	1962

***Note*:***VV Vespa velutina* group, *VAF Vespa analis fabricius* group, *VTD Vespa tropica ducalis* group, *VMS Vespa mandarinia smith* group

### Coding sequence domain prediction

The open reading frame (ORF) and coding domain sequence (CDS) of the wasps were predicted using the sequence information and reference structures obtained from ORFfinder. In all, 3,557,399 CDSs were predicted and clustered, including different types of ORFs (Additional file 3).

### Homology-based annotation of transcripts

**Fig. 2:**
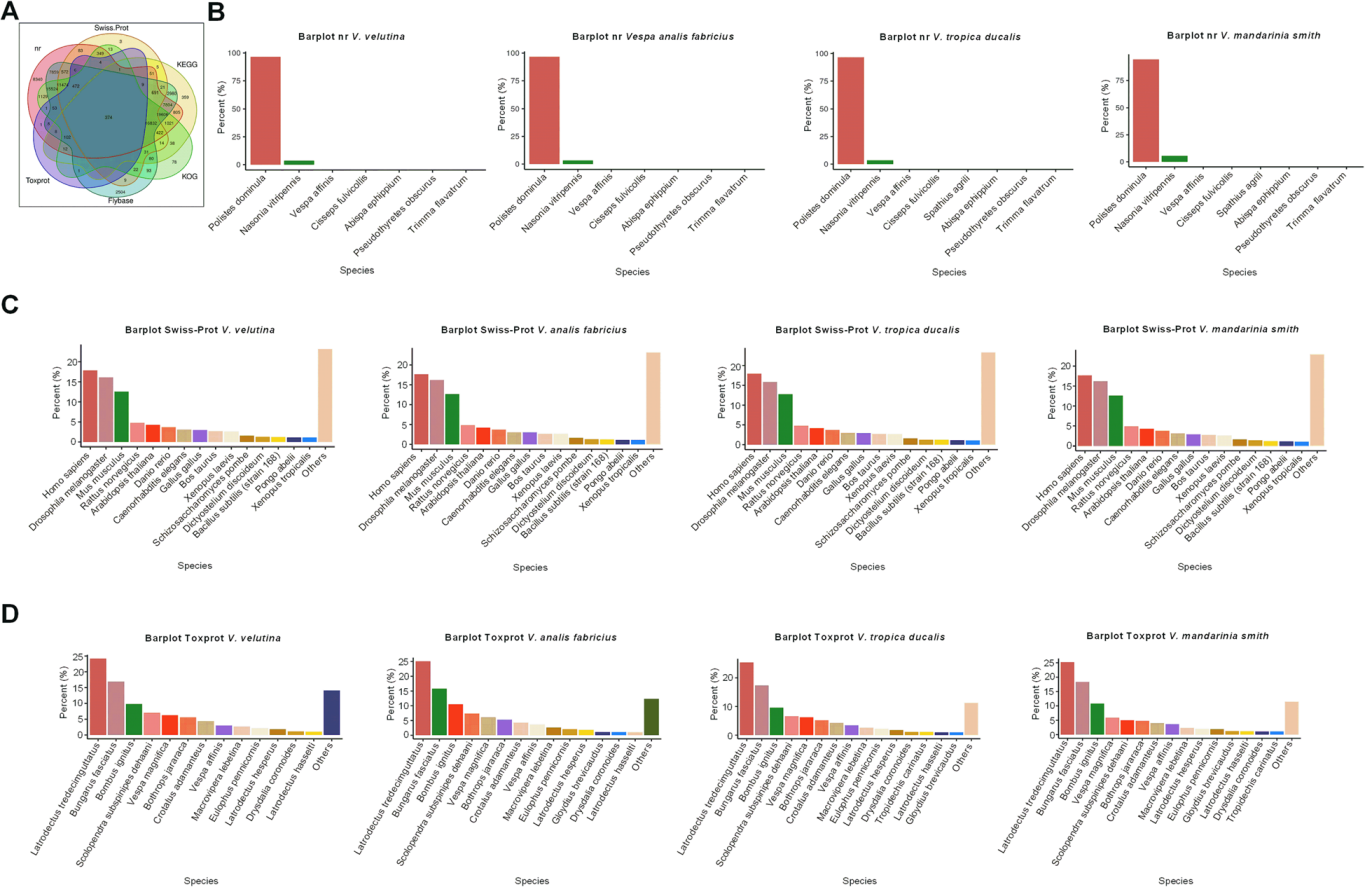
Homology-based annotation of transcripts. **a** The Venn diagram showing the overlap of unigenes annotated in Flybase, KEGG, KOG, nr, Swiss-Prot, and Tox-Prot databases. Annotation results of unigenes from the four wasp species of *V. velutina*; *V. analis fabricius*; *V. tropica ducalis*; *V. mandarinia smith* in (**b**) nr database. **c** Swiss-Prot database and (**d**) Tox-Prot database

The unigenes from the four different wasps were compared to the Flybase, KEGG, KOG, nr, Swiss-Prot, and Tox-Prot databases using BLASTX (E-value < 10^− 5^), and the results showed that 374 unigenes were annotated in all of these databases (Fig. 2a). Furthermore, for individual wasp species, *V. velutina*, *V. analis fabricius*, *V. tropica ducalis* and *V. mandarinia smith. V. mandarinia smith* had 304, 316, 315 and 332 unigenes annotated into all databases, respectively (Additional file 4). In the nr database, the species of the annotated homologous sequences of *V. velutina*, *V. analis fabricius*, *V. tropica ducalis* and *V. mandarinia smith* were mainly *Polistes dominula* (more than 90%), *Nasonia vitripennis* and *Vespa affinis* (Fig. 2b). In the Swiss-Prot database, the species hits of the annotated homologous sequences of *V. velutina*, *V. analis fabricius*, *V. tropica ducalis* and *V. mandarinia smith* were mainly *Homo sapiens*, *Drosophila melanogaster*, *Mus musculus* and *Rattus norvegicus* (Fig. 2c). Moreover, in the Tox-Prot database, the species of the annotated homologous sequences of *V. velutina*, *V. analis fabricius*, *V. tropica ducalis* and *V. mandarinia smith* were mainly *Latrodectus tredecimguttatus*, *Bungarus fasciatus*, *Bombus ignitus* and *Scolopendra subspinipes dehaani* (Fig. 2d). These results indicated that the unigenes of the four different wasps (*V. velutina*, *V. analis fabricius*, *V. tropica ducalis* and *V. mandarinia smith*) were annotated in the nr, Swiss-Prot and Tox-Prot database to obtain the similar species information.

**Fig. 3:**
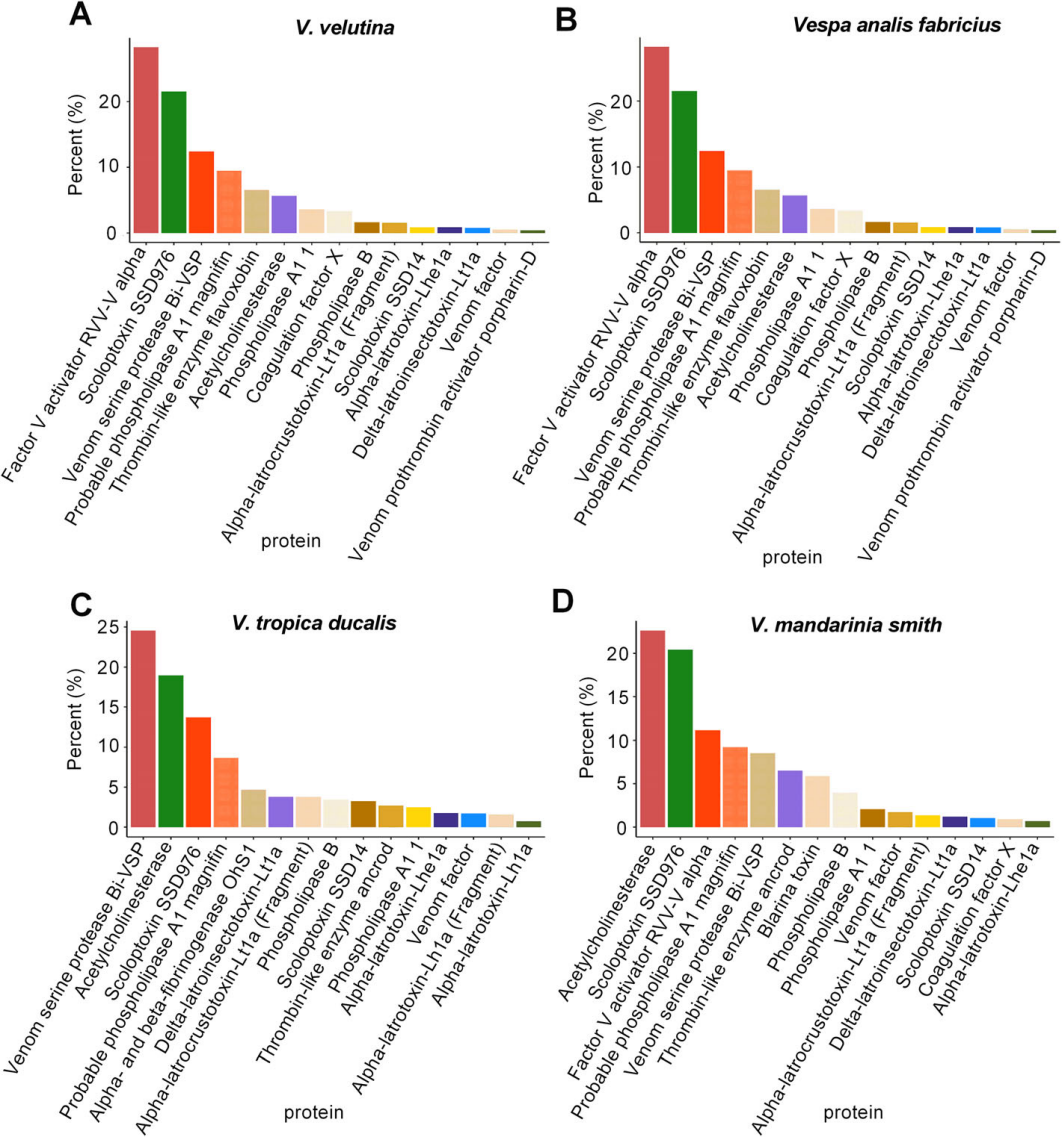
Number of top hits with significant homologous to the toxins in Tox-Prot. **a** Top hits in Tox-Prot for *Vespa velutina* group. **b** Top hits in Tox-Prot for *Vespa analis fabricius* group. **c** Top hits in Tox-Prot for *V. tropica ducalis* group. **d** Top hits in Tox-Prot for *V. mandarinia smith* group

We further plotted the classification of four species of wasp’s venom toxins by using a blastx search for Tox-Prot database (Fig. 3). The results showed that *V. velutina* group and *V. analis fabricius* group had similar classification of toxins, mainly composed of Factor V activator RVV-V alpha, Scoloptoxin SSD076, Venom serine protease Bi-VSP, Probable phospholipase A1 magnifin and Thrombin-like enzyme flavoxobin (Fig. 3a, b). Venom serine protease Bi-VSP, Acetylcholinesterase, Scoloptoxin SSD976, Probable phospholipase A1 magnifin, and Alpha-latrocrustotoxin-Lt1a (Fragment) accounted for a high proportion in the *V. tropica ducalis* group (Fig. 3c). In the *V. mandarinia smith* groups, the main annotated proteins were Acetylcholinesterase, Scoloptoxin SSD976, Factor V activator RVV-V alpha, Probable phospholipase A1 magnifin, and Venom serine protease Bi-VSP (Fig. 3d). These results indicated that the species and proportion of toxins contained in the four venom glands were different and may vary from species to species.

### GO enrichment analysis

**Fig. 4:**
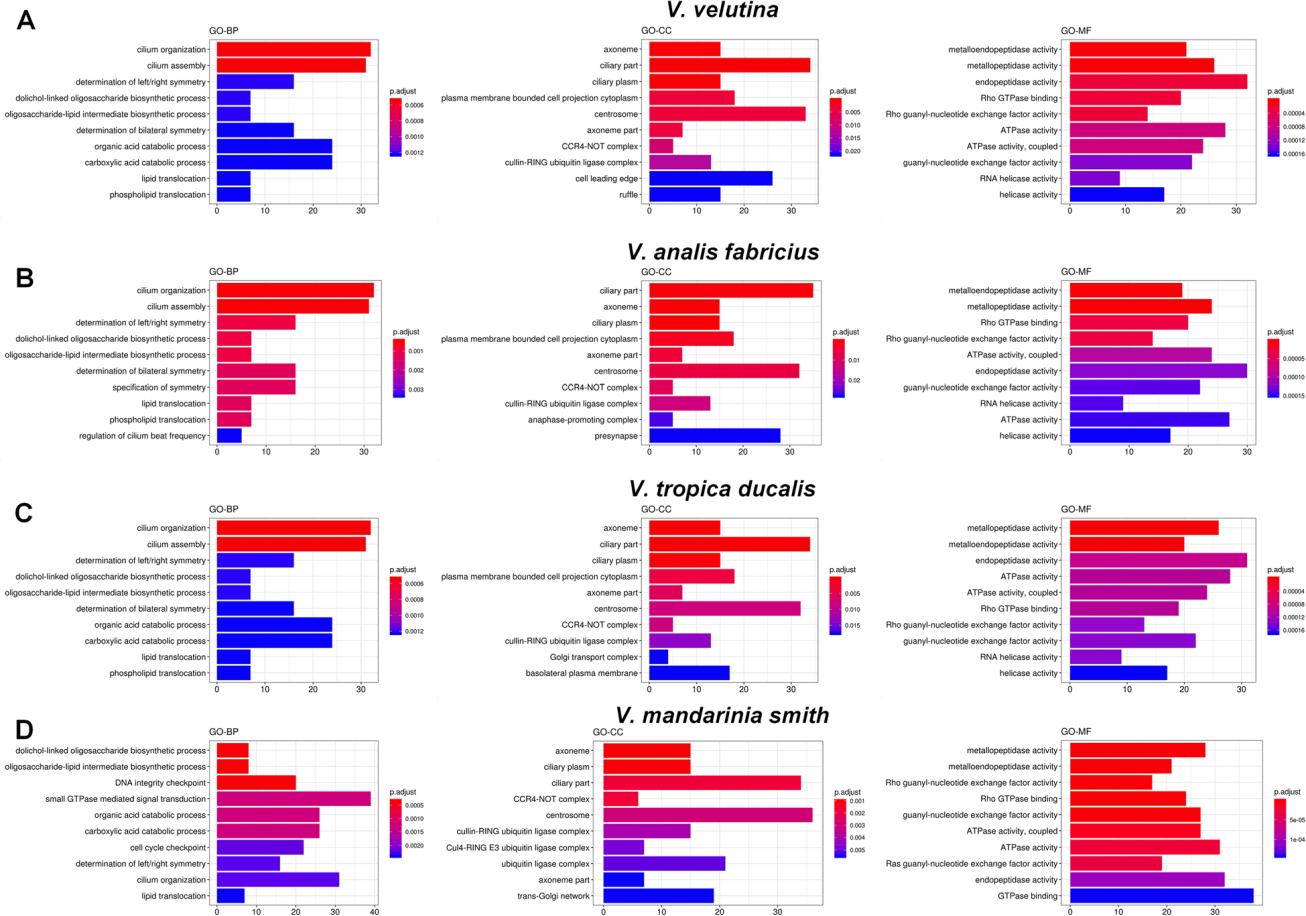
GO enrichment analysis of unigenes from the gland of each species. **a** GO enrichment analysis of unigenes from *V. velutina*. **b** GO enrichment analysis of unigenes from *V. analis fabricius*. **c** GO enrichment analysis of unigenes from *V. tropica ducalis*. **d** GO enrichment analysis of unigenes from *V. mandarinia smith* group. Only the top 10 GO-terms are displayed in the categories of biological process (GO-BP), cellular component (GO-CC) and molecular function (GO-MF)

The GO enrichment of unigenes of *V. velutina* group showed that 136 terms were enriched and contained 69 terms in BP, 38 in MF, and 29 CC (Additional file 5). As shown in Fig. 4a, cilium organization and cilium assemble were significantly enriched in BP. Axoneme, ciliary part, ciliary plasm, plasma membrane bounded cell projection cytoplasm, centrosome and axoneme part were terms significantly enriched in CC while metallopeptidase activity, metalloendopeptidase activity, endopeptidase activity, Rho GTPase binding, and Rho guanyl-nucleotide exchange factor activity were significantly enriched in MF terms (Additional file 5).

The GO enrichment of unigenes of *V. analis fabricius* group showed that 136 terms composed of 74 terms in BP, 36 in MF, and 26 in CC were enriched (Additional file 6). As shown in Fig. 4b, cilium organization and cilium assemble were significantly enriched in BP; ciliary part, axoneme, ciliary plasm, and plasma membrane bounded cell projection cytoplasm were significantly enriched in CC; and metallopeptidase activity, metalloendopeptidase activity, Rho GTPase binding, and Rho guanyl-nucleotide exchange factor activity were significantly enriched in MF (Additional file 6).

The GO enrichment of unigenes of *V. tropica ducalis* group showed that 136 terms were classified as BP (70 terms), MF (39 terms), and CC (17 terms) (Additional file 7). As shown in Fig. 4c, cilium organization and cilium assemble were significantly enriched in BP; axoneme, ciliary part, ciliary plasm, and plasma membrane bounded cell projection cytoplasm were significant enriched in CC; and metallopeptidase activity and metalloendopeptidase activity were significant enriched in MF (Additional file 7).

The GO enrichment of unigenes of *V. mandarinia smith* group showed that 166 terms were enriched and could be classified as BP (88 terms), MF (43 terms), and CC (35 terms) (Additional file 8). As shown in Fig. 4d, dolichol-linked oligosaccharide biosynthetic process, oligosaccharide-lipid intermediate biosynthetic process, and DNA integrity checkpoint were significantly enriched in BP. Axoneme, ciliary plasm, ciliary part and CCR4-NOT complex were significantly enriched in CC while metallopeptidase activity, metalloendopeptidase activity, Rho guanyl-nucleotide exchange factor activity, Rho GTPase binding, guanyl-nucleotide exchange factor activity, ATPase activity, coupled, and ATPase activity were significantly enriched in MF (Additional file 8).

**Fig. 5:**
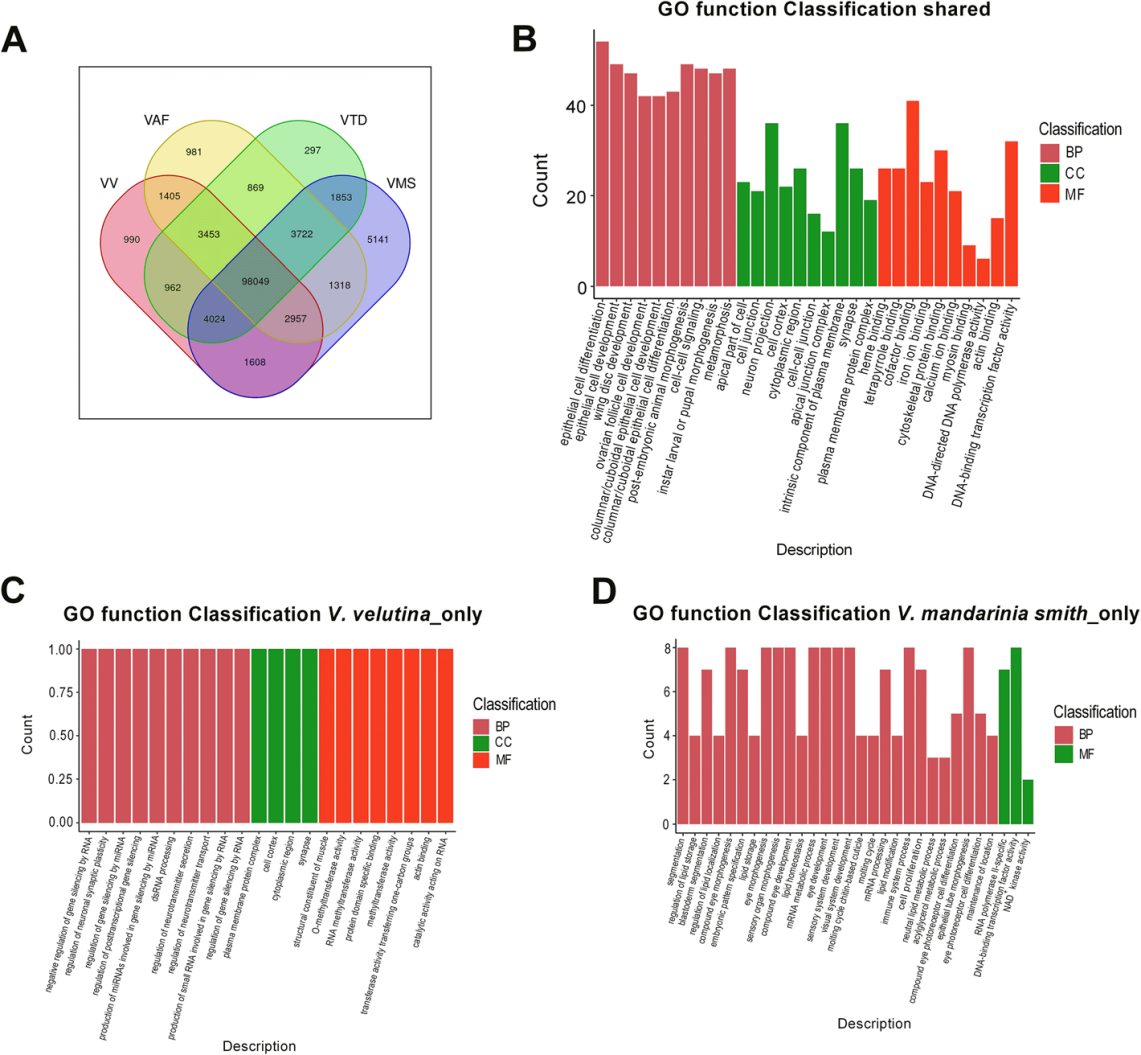
GO enrichment analysis of common and specific unigenes of the four wasp species. **a** The Venn diagram showing that the common and specific unigenes of *V. velutina (VV)*, *V. analis fabricius* (VAF), *V. tropica ducalis* (VTD) and *V. mandarinia smith* (VMS). **b** GO enrichment analysis of unigenes shared by the four wasp species. **c** GO enrichment analysis of unigenes specific to *V. velutina.***d** GO enrichment analysis of unigenes specific to *V. mandarinia smith*

Through the Venn diagram we found that 1608 unigenes were common to the four species of wasp (*V. velutina*, *V. analis fabricius*, *V. tropica ducalis* and *V. mandarinia smith*) (Fig. 5a). Additionally, as shown in Fig. 5a, the specific unigenes detected in *V. velutina*, *V. analis fabricius*, *V. tropica ducalis* and *V. mandarinia smith* were 990, 981, 297 and 5141, respectively (Fig. 5a). Among them, *V. mandarinia smith* had the most unique unigenes, indicating that *V. mandarinia smith* may have more genomic information than the other three species of wasps.

We further carried out GO enrichment analysis on the shared and specific unigenes of the four species of wasp. The GO enrichment of unigenes shared by the four species of wasp showed that 1089 GO terms (904 in BP, 74 in MF, and 111 CC) could be enriched (Additional file 9). As shown in Fig. 5b, epithelial cell differentiation, epithelial cell development, wing disc development, ovarian follicle cell development and columnar/cuboidal epithelial cell development were terms significantly enriched in BP; apical part of cell, cell junction, neuron projection, and cell cortex were terms significantly enriched in CC; heme binding, tetrapyrrole binding, cofactor binding and iron ion binding were significantly enriched in MF (Additional file 9).

GO enrichment analysis of unigenes specific to each of the four wasp species showed that only *V. velutina* and *V. mandarinia smith* groups had enrichment data. The GO enrichment of unigenes specific to *V. velutina* showed that 45 GO terms were enriched and included 33 terms in BP, 8 in MF, and 4 in CC (Additional file 10). As shown in Fig. 5c, negative regulation of gene silencing by RNA, regulation of neuronal synaptic plasticity, regulation of gene silencing by miRNA, regulation of posttranscriptional gene silencing, and production of miRNAs involved in gene silencing by miRNA were significantly enriched in BP while plasma membrane protein complex, cell cortex, cytoplasmic region, and synapse were terms significantly enriched in CC. Terms such as structural constituent of muscle, O-methyltransferase activity, RNA methyltransferase activity and protein domain specific binding were those significantly enriched in MF (Additional file 10).

The GO enrichment of unigenes specific to *V. mandarinia smith* showed that 30 terms (27 in BP and 3 in MF) were enriched (Additional file 11). As shown in Fig. 5d, segmentation, regulation of lipid storage, blastoderm segmentation, regulation of lipid localization, and compound eye morphogenesis were significant enriched in BP; RNA polymerase II-specific, DNA-binding transcription factor activity, and NAD^+^ kinase activity were significantly enriched in MF (Additional file 11).

### SSR and SNP analysis

**Fig. 6:**
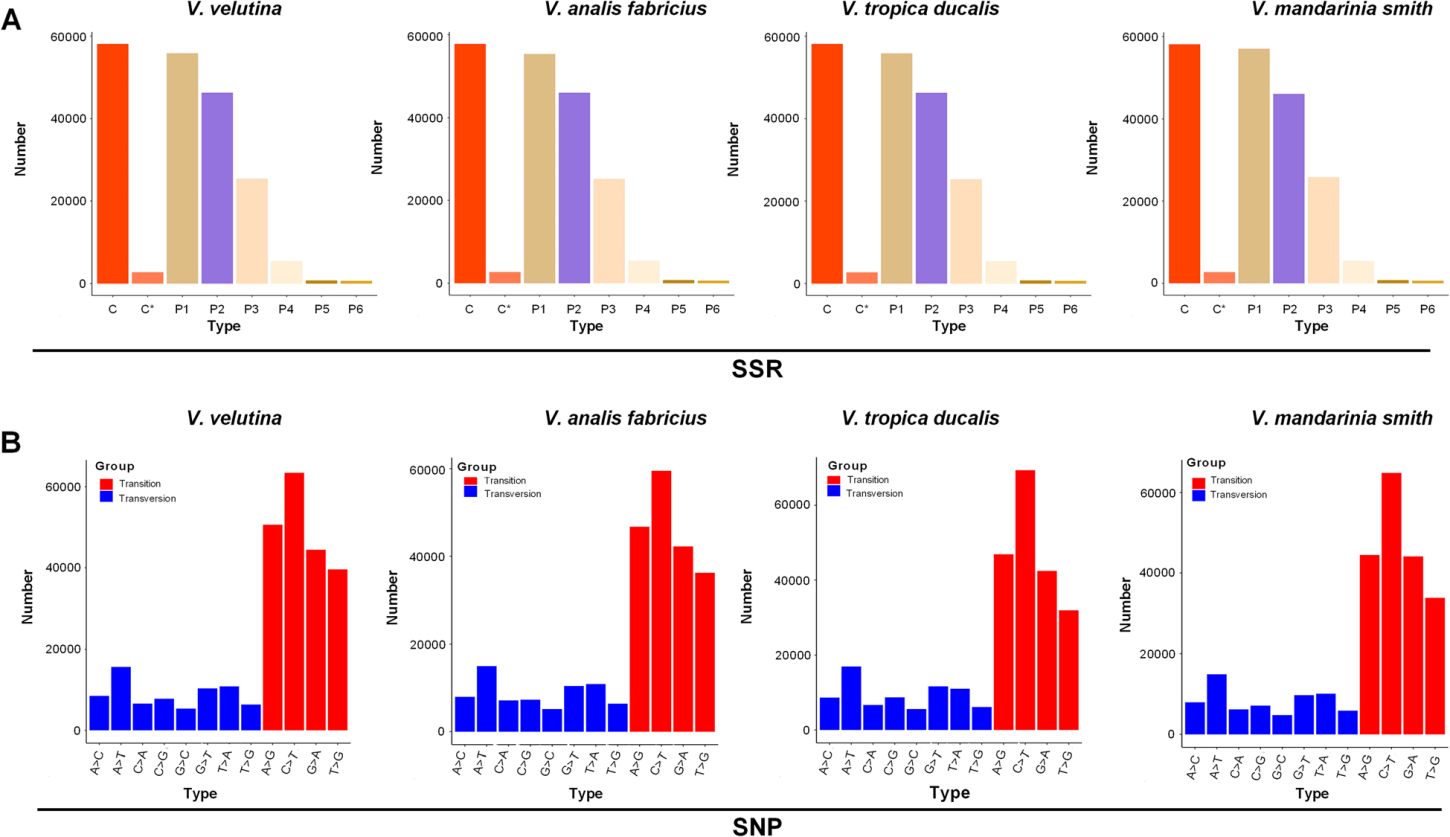
Identification of molecular markers in the venom gland of the four wasp species. **a** Identification of SSRs in the venom gland of *V. velutina, V. analis fabricius*, *V. tropica ducalis and V. mandarinia smith*. **b** Identification of SNPs in the venom gland of *V. velutina, V. analis fabricius*, *V. tropica ducalis and V. mandarinia smith*

SSR analysis was performed on the four species of wasp transcriptome samples using the MIcroSAtellite identification tool (MISA) software. The results showed that 195,330, 193,994, 195,152 and 196,691 SSRs were detected in the *V. velutina*, *V. analis fabricius*, *V. tropica ducalis* and *V. mandarinia smith* transcriptome samples, respectively, for a total of 8 SSR types, namely c, c*, p1, p2, p3, p4, p5 and p6 (Fig. 6a). Among them, c-type, p1 type, p2 type and p3 type were the most commonly found SSR types in the transcriptome of the four wasp species.

SNP variants were analyzed separately according to the species, and these mutations could result in synonymous, nonsynonymous and even some frame-shift in transcription process. The results showed that 269,585 (198,185 for transition and 71,400 for transversion), 254,863 (184,699 for transition and 70,164 for transversion), 266,084 (190,664 for transition and 75,420 for transversion) and 253,901 (187,321 for transition and 66,580 for transversion) SNPs were identified in the *V. velutina*, *V. analis fabricius*, *V. tropica ducalis* and *V. mandarinia smith* transcriptomes, respectively. As shown in Fig. 6b, the number of transition types (C- > T, A- > G, G- > A and T- > C) was significantly higher than the transversion types (A- > T, G- > T, T- > A, C- > G, A- > G, C- > A, T- > G and G- > C), where C- > T type was the most abundant of all four species of wasp samples. There were differences in the distribution of SNPs among the four species of wasp, and *V. tropica ducalis* had a higher C- > T type whereas *V. velutina* had a higher T- > C type.

## Discussion

With the development of NGS technology and bioinformatics methods, RNA sequencing, unlike traditional Sanger sequencing, can yield high-throughput and high-quality transcriptome data, which has been introduced as a vital tool for scientific researches [41–44]. To the best of our knowledge, this is the first study to develop a full-scale map of *V. tropica ducalis*, *V. analis fabricius*, and the specific wasp subfamily *V. mandarinia smith*. More details and information on *V. velutina* were also obtained. The transcriptome of the venom glands of the four wasp species were analyzed using the Illumina Hiseq4000 RNA-seq platform. The assembled contigs were annotated and blasted into the published database of fruit fly, which revealed 98,716 CDSs, of which 67,076 annotated genes and 24,539 unannotated genes were aligned. Unannotated unigenes probably belong to untranslated regions, non-coding RNA, novel genes and short sequences without protein domains or assembly errors, and suggest that the wasp venom likely has some uncharacterized sequences. We further found that 1608 unigenes were detected in all four species of wasp (*V. velutina*, *V. analis fabricius*, *V. tropica ducalis* and *V. mandarinia smith*). The specific unigenes detected in *V. velutina*, *V. analis fabricius*, *V. tropica ducalis* and *V. mandarinia smith* were 990, 981, 297 and 5141, respectively. Among them, *V. mandarinia smith* had the higher number of specific contigs, indicating that *V. mandarinia smith* may have more genomic information than the other three species of wasps. Thus, our study might directly or indirectly promote or contribute to the completion of the sequencing of wasp venom.

The unigenes of the four wasp species were searched for the Tox-Prot database by blastx to annotate the venom protein. We found that these unigenes were annotated as Factor V activator RVV-V alpha, Acetylcholinesterase, Venom serine protease Bi-VSP, Scoloptoxin SSD076, Probable phospholipase A1 magnifin, Thrombin-like enzyme flavoxobin, etc. Studies have shown that factor V activator and thrombin-like enzyme are involved in the blood coagulation cascade, which has been reported in the *V. velutina* venom gland, but rarely reported in other Hymenoptera insects [14]. The role of bee venom serine protease is reported only in *Bombus ignitus* as a prophenoloxidase activating factor, triggering a cascade of phenoloxidase, which acts like a snake venom serine protease and has fibrin (ogen) olytic activity [45, 46]. Previous studies showed that phospholipase A1 magnifin, identified from the *Vespa affinis* venom gland, is involved in serum IgE responses as a major allergenic protein [47]. Acetylcholinesterase has been found in the venom of *V. velutina*, which plays key roles in neurotransmission through inactivating the acetylcholine, ultimately resulting in paralysis of the prey [14, 48]. These data provide clinical support for the potential use of venom gland proteins and is expected to be used for developing specific anti-bee venom similar to anti-venom serum.

Due to the influence of factors such as individual differences, sex differences and geographical environment differences, it is easy to confuse similar species in the traditional identification of wasps. DNA barcoding approach was found to be an efficient method for classifying wasp species. The mitochondrial COI gene has the characteristics of high evolution rate, obvious interspecific variation and relatively conservative species, and has been widely used as an effective DNA barcoding for species classification and evolution analysis [49, 50]. The success rate of DNA barcoding method was nearly 100%, as verified by the RNA-Seq process. Similar barcoding procedures have been used in snakes [31], spiders [51], and parasitoid wasps [52], showing the feasibility and efficiency of this method in distinguishing specific DNA sequences in the venom. In this study, based on COI sequence identification, the NJ tree indicated that the *V. analis fabricius* − 1 and − 7 COI sequences were divergent from the other six COI sequences. They were homologous and all clustered in the same big branch, indicating that they were from the same subspecies, the *V. analis fabricius* − 1 and − 7 COI might have undergone different changes under environmental and evolutionary pressures. It also revealed the unique lineage of these individuals, and the clustering information clarified the differences and similarities in the molecular sequences.

The traditional development method of SSR molecular markers is to construct DNA libraries for screening, which is costly and inefficient, and the application of high-throughput sequencing technology brings down to large-scale screening of SSR molecular markers [53]. SSR is a significant molecular marker for specific functional gene studies, genetic linkage map construction, as well as evolutionary analysis of plants and animals [54, 55]. In this study, 8 types of SSR were detected in the four wasp species, of which c, p1, p2 and p3 were the most common SSR types in the four species of wasp. Moreover, SNP molecular marker technology is the third generation of molecular marker technology after SSR. It can fully reflect the genetic and variational levels of the population by studying its distribution in the biological genome. In molecular evolution, it is common for nucleotide transitions to be several times higher than transversions. In Stoltzfus’ et al. study, they integrated eight studies about the fitness for replacement mutations and found that transitions were more suitable than transversions at a 53% chance (95% CI 50 to 56). It confirmed that the transitions conservative evolutionary frequency of crossings was increased [56]. In this study, we found that the number of transition types was significantly higher than the types of transversion, and the distribution of SNPs among the four species of wasp was also different. These data are valuable for further molecular researches of the wasps. Furthermore, the valuable genetic information (SSRs and SNPs) might be useful for research on functional genes by using genomic and proteomic tools, and can be developed to help conserve wasp species in their preferred habitat. In this study, we found that a number of genes were shared by the four species while other genes were specific to each wasp species. In addition, most of the genes could not be annotated in the databases. These observations indicated that there is a large number of genes in the venom gland of wasp species that need to be identified. Identifying these genes will further our understanding on the toxins produced by these species. This will play a significant role in the applications of wasp species to pest control and medical purposes. Until then, the annotated genes discovered in the present study will be useful for current studies but the validation of these genes by additional molecular biology techniques and functional analysis by experimentation will be performed in our future studies.

## Conclusions

In this study, the complete transcriptome of four wasps (*V. velutina*, *V. mandarinia smith*, *V. analis fabricius*, and *V. tropica ducalis*) was sequenced using the Illumina HiSeq 4000 NGS platform. The sequences were assembled into meaningful unigene sequences by using the de novo assembler Trinity and the clustering tool TGICL. The unigene sequences were annotated into publicly available protein sequence databases for putative functional attributes, especially their assignment to GO categories. This information provides clinical support for the potential use of venom gland proteins and is expected to be used for developing specific anti-bee venom similar to anti-venom serum. In addition, we obtained a set of reliable SSR and SNP markers from the unigene sequences. This valuable genetic information should be useful for studying functional genes by using genomic and proteomic tools.

## Methods

### Insects

Four social wasp species with different phenotypes were collected from wild populations in Baoshan (Yunnan province, China). All wasps were collected directly from the nests by using forceps, preserved immediately in RNAlater (Ambion, Invitrogen, Applied Biosystems) and stored at − 20 °C until analysis. At the very beginning, wasps were classified according to their morphological differences. Wasps with yellow feet were classified into the *V. velutina* group. Wasps with a golden ring were classified into the *V. mandarinia smith* group. Wasps with a black rump were classified into the *V. tropica ducalis* group while wasps with thick skin were classified into the *V. analis fabricius* group. The “Wildlife Protection Law of the People’s Republic of China” stipulates that:

- Article 2: The wildlife protected by this Law refers to the precious and endangered terrestrial and aquatic wildlife and terrestrial wildlife with important ecological, scientific and social values.
- Article 21: It is forbidden to hunt, kill or kill wildlife under special state protection.

After investigation, we found that wasp species are not included in the “National List of Key Wildlife Protection”, and in fact, there is a large number of wasps species, which are ubiquitous and are not endangered species, so they are not considered as “wild animals” protected by the “Wild Animal Protection Law”. Therefore, no ethical approval nor permission was required for collecting and carrying out experiments on the four wasp species (*V. velutina*, *V. tropica ducalis*, *V. analis fabricius* and *V. mandarinia smith*), which are neither endangered nor protected species.

### DNA barcoding

Proteinase K was used to digest the preserved tissues followed by addition with phenol-chloroform to extract the total genomic DNA. The concentration was determined using a UV spectrophotometer. DNA samples were diluted with concentrations ranging from 30.0 to 80.0 ng/μl for the next step. Subsequently, based on previous studies [57], the mitochondrial cytochrome C oxidase submit I (COI) gene was amplified using the universal primers HC02198 and LC01490 in a reaction volume of 25 μl containing 2.5 μl of 10× PCR buffer, 1.5 μl of 10 μM primer, 2 μl of dNTP, 0.25 μl of Taq polymerase, 2 μl of genomic DNA, and 16.75 μl of PCR water. The PCR cycling profiles were as follows: 5 min initial denaturation at 94 °C, followed by 5 cycles of pre-amplification for 30 s at 94 °C, annealing for 30 s at 50 °C to 45 °C, and extension for 30 s at 72 °C. Next, 35 cycles of amplification were run at 94 °C for 30 s, 45 °C for 30 s, and 72 °C for 30 s, and a final extension for 10 min at 72 °C. The PCR products were extracted and purified. Subsequently, sequencing was performed by Sanger sequencing.

The nucleotide sequences were aligned in MEGA 6.0 [58] with default parameters, which pairwise and multiple alignment with the gap opening penalty was both 10 and the gap extension penalty was 0.1 and 0.2, respectively. The formulation of CR = ∑[σi/μi]2/(n-3), where n is the number of sequences, was used to generate a 95% confidence interval. BLAST search was performed by inputting the FASTA file in the nucleotide database in GenBank (https://blast.ncbi.nlm.nih.gov/Blast.cgi). Then we obtained the gene sequence with high homology and download it to FASTA format. Moreover, we used CLUSTAL X to perform multiple sequence alignment to generate a guide tree file (DND file). Finally, based on the DND file that generated by CLUSTAL X, a neighbor-joining tree (NJ-tree) was generated using MEGA 6.0, and the intergenic, interspecific, and intraspecific sequence distances were estimated. All of the insertions and deletions were tested by the bootstrap method with 1000 replicates, and the nucleotide sequences with homologies of 100% were isolated as representative to construct the phylogenetic tree.

### RNA extraction, library construction, and sequencing

Total RNAs were isolated from the venom gland tissues by using RNeasy Micro Kit (Qiagen, France) according to the manufacturer’s instructions. Sequencing and cDNA library preparation were performed using Beckman Coulter Genomics services (http://www.beckmangenomics.com/). The mRNA extracted from the venom gland tissues was reverse transcribed into cDNA and amplified using the Ovation RNA-Seq System V2 kit (NuGEN Technologies Inc., USA). After cDNA fragmentation, end-repair and purification were performed using the Agencourt AMPure XP kit (Agencourt Bioscience, Beckman Coulter, San Carlos, CA, USA), and TruSeq sequencing adapters (Illumina, USA) were ligated to cDNA fragments. Finally, the library was PCR-amplified (14 cycles) to about 30 ng/μl by using a high-fidelity DNA polymerase (Beckman Genomics, USA). Illumina TruSeq adapters were ligated to the 5′ and 3′ ends of the cDNA of both samples. The cDNA was finally amplified by PCR using a proofreading enzyme (Beckman Genomics, USA). For Illumina sequencing, the cDNA samples were fractionated on agarose gels ranging in the size from 300 to 500 bp. PCR amplification was designed for TruSeq sequencing using the HiSeq4000 technology according to the instructions of Illumina.

### Sequence filtering, de novo assembly, and assembly validation

The raw sequencing data was assessed using FAST-QC (http://www.bioinformatics.babraham.ac.uk/projects/fastqc/), including quality distribution of nucleotides, position-specific sequencing quality, GC content, PCR duplication proportion, and kmer frequency. The quality score was set over 30 to obtain an accuracy of 99.9%. The filtered reads for each sample were merged and de novo assembled using Trinity software [43]. The operating parameters of Trinity program were: k-mer = 25, the minimum k-mer coverage = 2, the maximum length expected between the pair of fragments = 500, the minimum overlap with the reading of the transcript PE = 75 and the maximum number of readings anchored in a single figure = 200,000 [59]. After that, three software modules (Inchworm, Chrysalis, and Butterfly) were applied using Trinity and the joint sequences were clustered using Cap3 [60]. CAP3 used base quality for constructing the alignment of reads and generating a consistent sequence for each Contig. The distance recognized by CAP3 and the inserted value generally differ by 1000 to 1500 bp, and the default quality value of each base is 10 [60]. The fastq type reads and paired reads were assembled into contigs, generating a final reference sequence, which was used for functional annotation analysis.

### Transcriptome annotation and analysis

Unigene annotation was conducted using a series of bioinformatics approaches. The published databases, including NCBI non-redundant protein (nr) databases (http://www.ncbi.nlm.nih.gov/), the Gene ontology database (http://www.geneontology.org/), the KEGG database (http://www.genome.jp/kegg/), the Swiss-Prot database (http://www.uniprot.org/keywords/), and the Tox-Prot database (http://www.uniprot.org/program/Toxins) were used to search for unigene annotations and sequence alignments. Certain algorithms were performed using BLASTX (parameter values showed in Tables 2 and 3) with the E-value cutoff of 1E-5 for pathway assignment, domain/family comparison, and mapping. The molecular function (MF), biological process (BP), including GO annotation and classification, and cellular component (CC) ontologies were conducted using Blast2GO program. Furthermore, the unigenes were equally mapped to the Flybase, KEGG, KOG, Swiss-Prot, and Tox-Prot (animal toxin database) except nr databases for the prediction and classification of potential functions by using the BWA-MEM algorithm of BWA software. The nr database with huge data and selected by limiting the BLAST+ search by taxonomy (−taxidlist wasplst.txt) to reduce the scope of search efficiency and calculation time.

**Table 2:** The parameters list for blastx software running in diverse databases

Item	Parameter value (Flybase, KEGG, KOG, Swiss-Prot, and Tox-Prot databases)
Query	UniGene.fa
Db	index/uniprot_sprot.fasta
Out	swiss_prot_result.blast-its-1.xl
Evalue	1e-5
Max_target_seqs	1
outfmt	“6 qseqid sseqid salltitles qlen qstart qend slen sstart send evalue bitscore pident staxids qcovs”
num_threads	40

**Table 3:** The parameters list for blastx software running in nr database

Item	Parameter value (nr database)
Query	4.UniGene.fa
Db	/home/tmp_database/nr_v5/nr_v5
taxidlist	wasplst.txt
Out	nr_result.blast-its--1.xl
Evalue	1e-5
Max_target_seqs	1
outfmt	“6 qseqid sseqid salltitles qlen qstart qend slen sstart send evalue bitscore pident staxids qcovs”
num_threads	40

### GO enrichment analysis

GO analysis was applied to analyze the main function of unigenes according to Gene Ontology [61]. We computed the *P*-values for the GOs of all unigenes. Within the significant category, the relative enrichment was given as follows: Re = (n_f_/n)/(N_f_/N), where n_f_ is the number of unigenes in a particular category, n is the total number of genes in the same category, N_f_ is the number of unigenes in the entire array, and N is the total number of genes in the array.

### Genomic analysis for putative molecular markers

More information on the genome was obtained by performing Single Sequence Repeat (SSR) and Single Nucleotide Polymorphisms (SNP) analyses. The MIcroSAtellite identification tool, a script from perl for identifying SSRs, was applied for detecting one to six bases of repeat units. The density and distribution of different SSR types were determined in the whole genome. Primer 3 (http://primer3.sourceforge.net/) was run under default parameters to design SSR primers. Moreover, GATK software was used to align the exon reads and reference sequence and SNPs were picked [62]. For information about GATK’s best practices for SNP calls, please see the GATK’s official website (http://www.broadinstitute.org/gatk/guide/best-practices).

## Supplementary information

**Additional file 1.** The results of homologous alignment of seven vespa groups.

**Additional file 2.** The statistics of the sequencing data of 12 wasp individuals.

**Additional file 3.** Results of ORF prediction using ORFfinder.

**Additional file 4 **Venn diagram showing the intersection of anaotation results from blast against Flybase, KEGG, KOG, nr, Swiss-Prot and Tox-Prot database. **(A)** The intersection of the significant hits for *V. velutina.***(B)** The intersection of the significant hits for *V. analis fabricius.***(C)** The intersection of the significant hits for *V. tropica ducalis***(D)** The intersection of the significant hits for *V. mandarinia smith*.

**Additional file 5 **The GO enrichment of unigenes from venom gland of *V. velutina*.

**Additional file 6 **The GO enrichment of unigenes from venom gland of *V. analis fabricius*.

**Additional file 7 **The GO enrichment of unigenes from venom gland of *V. tropica ducalis*.

**Additional file 8 **The GO enrichment of unigenes from venom gland of *V. mandarinia smith*.

**Additional file 9.** The GO enrichment of unigenes shared by the venom gland of the four wasp species.

**Additional file 10 **The GO enrichment of unigenes specific to the venom gland of *V. velutina*.

**Additional file 11 **The GO enrichment of unigenes specific to the venom gland of *V. mandarinia smith*.

## Data Availability

All data generated or analyzed during this study are included in this published article and its supplemental files. Twelve transcriptome sequencing data for four species of wasp have been uploaded to the NCBI Sequence Read Archive (SRA, http://www.ncbi.nlm.nih.gov/Traces/sra) database under the accession numbers: SRR9157365-SRR9157376.

## Abbreviations

COI: Cytochrome C oxidase submit I
NGS: Next-generation sequencing
SNP: Single Nucleotide Polymorphisms
VAAM: Vespa amino acid mixtures
